# Feasibility of postoperative spine stereotactic body radiation therapy in proximity of carbon and titanium hybrid implants using a robotic radiotherapy device

**DOI:** 10.1186/s13014-022-02058-7

**Published:** 2022-05-12

**Authors:** Dominik Henzen, Daniel Schmidhalter, Gian Guyer, Anna Stenger-Weisser, Ekin Ermiş, Robert Poel, Moritz Caspar Deml, Michael Karl Fix, Peter Manser, Daniel Matthias Aebersold, Hossein Hemmatazad

**Affiliations:** 1 Division of Medical Radiation Physics and Department of Radiation Oncology, Inselspital, Bern University Hospital, University of Bern, Bern, Switzerland; 2 Department of Orthopedic Surgery and Traumatology, Inselspital, Bern University Hospital, University of Bern, Bern, Switzerland

**Keywords:** Postoperative SBRT, Spine metastasis, CFP-T implants, Cyberknife, Dosimetry analysis

## Abstract

**Background and purpose:**

To assess the feasibility of postoperative stereotactic body radiation therapy (SBRT) for patients with hybrid implants consisting of carbon fiber reinforced polyetheretherketone and titanium (CFP-T) using CyberKnife.

**Materials and methods:**

All essential steps within a radiation therapy (RT) workflow were evaluated. First, the contouring process of target volumes and organs at risk (OAR) was done for patients with CFP-T implants. Second, after RT-planning, the accuracy of the calculated dose distributions was tested in a slab phantom and an anthropomorphic phantom using film dosimetry. As a third step, the accuracy of the mandatory image guided radiation therapy (IGRT) including automatic matching was assessed using the anthropomorphic phantom. For this goal, a standard quality assurance (QA) test was modified to carry out its IGRT part in presence of CFP-T implants.

**Results:**

Using CFP-T implants, target volumes could precisely delineated. There was no need for compromising the contours to overcome artifact obstacles. Differences between measured and calculated dose values were below 11% for the slab phantom, and at least 95% of the voxels were within 5% dose difference. The comparisons for the anthropomorphic phantom showed a gamma-passing rate (5%, 1 mm) of at least 97%. Additionally the test results with and without CFP-T implants were comparable. No issues concerning the IGRT were detected. The modified machine QA test resulted in a targeting error of 0.71 mm, which corresponds to the results of the unmodified standard tests.

**Conclusion:**

Dose calculation and delivery of postoperative spine SBRT is feasible in proximity of CFP-T implants using a CyberKnife system.

## Introduction

Bone metastases are common in cancer patients and spinal column is involved in approximately two third of osseous metastases [1]. The treatment options for spine metastases are surgery, radiation therapy (RT), systemic therapy or a multimodality approach using a combination of these therapies. Spine metastases could cause mild to severe pain, pathologic fractures and neurological deficits limiting daily functions and deteriorating the performance status. In case of spinal instability, malignant epidural spinal cord compression (MESCC) with or without neurological symptoms and pathologic vertebral compression fracture (VCF), surgery is the standard of care followed by adjuvant conventional radiation therapy [2]. As for de novo spinal metastases [3–5], the oncological outcomes are not satisfactory after postoperative conventional RT (cRT), in particular the rate of local control (LC) remains low by 30–40% at 1 year [6–8]. Therefore, the number of patients who need further treatments after cRT is notable as they develop recurrent pain or tumor progression. As there is a rapid rise in development and implication of new systemic treatments such as targeted therapies and immunotherapy in recent years, the proportion of cancer survivors is increasing with the urgent need for more effective local treatments to improve the health related quality of life (HRQOL) [9]. On the other hand, advanced diagnostic imaging modalities are nowadays increasingly available, thus the metastatic sites are better recognized for local treatment as well as in follow-up and re-treatment in case of tumor/symptom progression [10]. Considering extended life expectancy for patients with malignancies in recent years and with the focus on RT, stereotactic body radiation therapy (SBRT) has become a substantial part of cancer treatment, especially in oligo-metastatic settings [11]. Several retrospective studies as well as some prospective data show outstanding local control after SBRT for intact spinal metastases [12–15]. Fewer publications reported excellent oncological outcomes in postoperative setting using SBRT for spinal lesions [2, 16, 17].

The widely used metal implants, mostly made of titanium, offer a reliable stability after spinal surgical interventions [18]. However, they are associated with several drawbacks regarding subsequent RT. The pure titanium implants make notable artifacts on computer tomography (CT) images, which are used to generate RT plans. These artifacts may lead to uncertainties in delineation of target volumes and organs at risk (OAR). For the RT treatment planning, the high density regions could cause inaccuracies in dose calculation [19]. Furthermore, for image guided radiation therapy (IGRT), high quality image information is a key requirement for accurate patient positioning. This is even more pronounced in SBRT were high dose gradients are present.

Besides titanium implants, the usage of carbon-fiber-composites in orthopedic implants goes back to past decades [20, 21]. In addition to their excellent biological and mechanical characteristics, CFP implants reduce artifacts on radiological images and decrease ionizing radiation absorption compared to pure titanium implants, thus support accurate RT planning and delivery [22]. However, the mentioned study only investigated the perturbation effects of CFP implants on radiation dose distributions in water phantom and did not show the feasibility of SBRT including its associated workflows. Furthermore, these implants simplify the radiological assessment in the postoperative setting and help the clinicians for a better recognition and treatment of local failures. Considering the fact that nowadays cancer patients have better survival than before, local failures should be diagnosed in time, as patients could profit from an adjuvant treatment. Therefore, there is an increasing clinical interest to use orthopedic implants, which are at least partly made of radiolucent CFP to support the whole RT process and do a better follow up for such patients with spinal metastases. In recent years and in line with the interest mentioned above, the department of orthopedic surgery in our university hospital applies CFP-T implants routinely for stabilizing vertebral column in patients with spinal metastases. Therefore, we performed our measurements with the same implants to simulate the actual clinical situation.

The current study assesses the planning and delivery feasibility of postoperative SBRT for spinal metastases in proximity of CFP-T hybrid implants using a Cyberknife M6 system (CK) (Accuray, Sunnyvale, CA, US).

## Materials and methods

Essential steps within a RT course were analyzed for phantoms and patients with CFP-T implants.

### Phantom and patient data

**Fig. 1:**
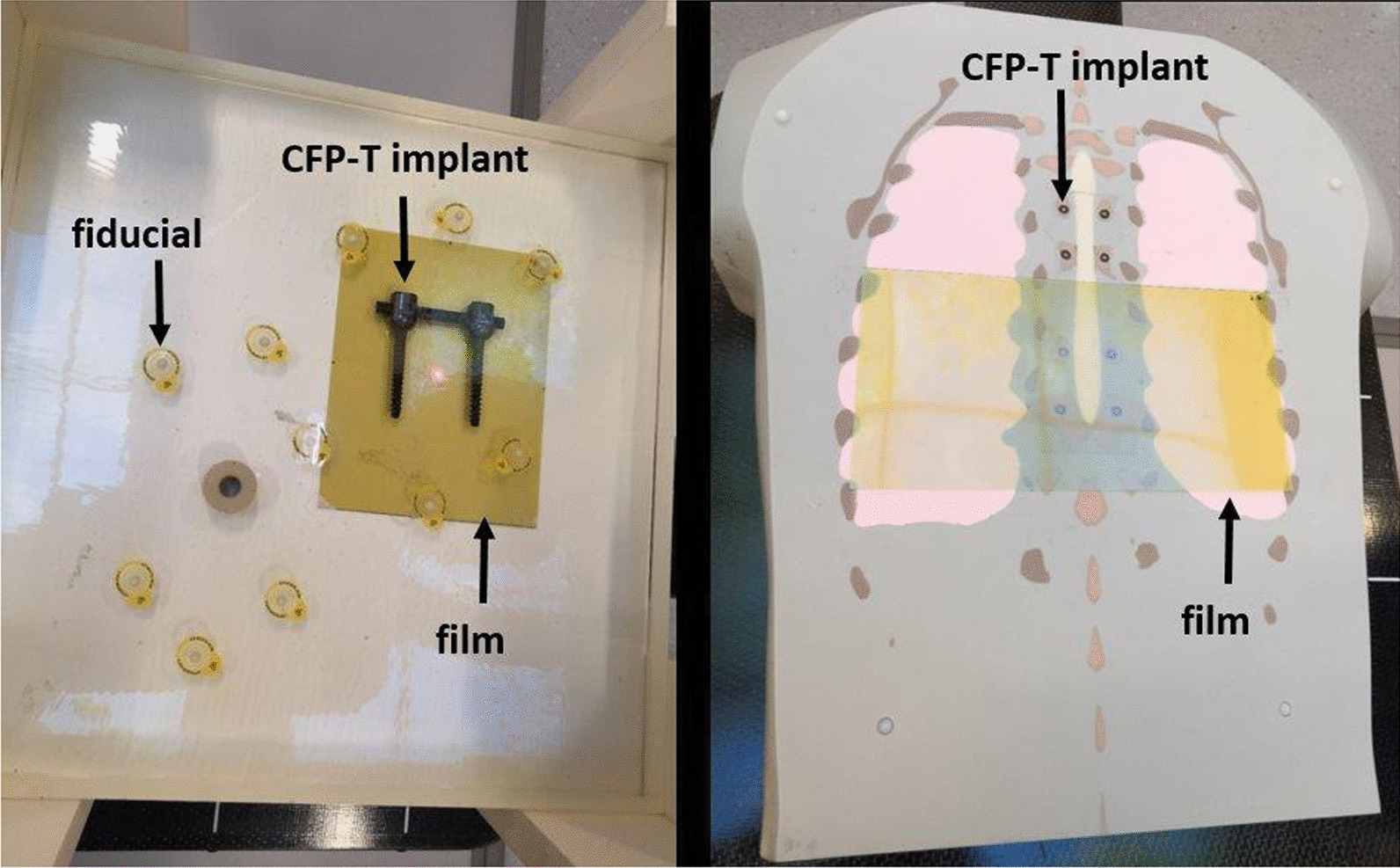
Image of the slab phantom (left) and the anthropomorphic phantom (right). The left picture shows the molded slab on the solid water phantom. The hybrid CFP-T (black) as well as the fiducials (inside the yellow circles) are visible. On the right side, the disassembled anthropomorphic phantom with the CFP-T implant is shown. The gafchromic films in yellow on both images indicate the area where measurements were carried out

A slab phantom was created by molding a CFP-T implant (Icotec AG, Altstätten, Switzerland) and numerous fiducial markers into epoxy resin (Fig. 1left). The resulting slab can be added to the commercially available RW3 solid water phantom (PTW, Freiburg, Germany). This offers the flexibility to change the depth of the implant as well as carrying out measurements at multiple distally located planes. For this work the CFP-T implant was positioned at a depth 4.5 cm and measurements were enabled at 0 mm, 2 mm, 10 mm and 50 mm distance distal to the slab with the CFP-T implant present.

**Fig. 2:**
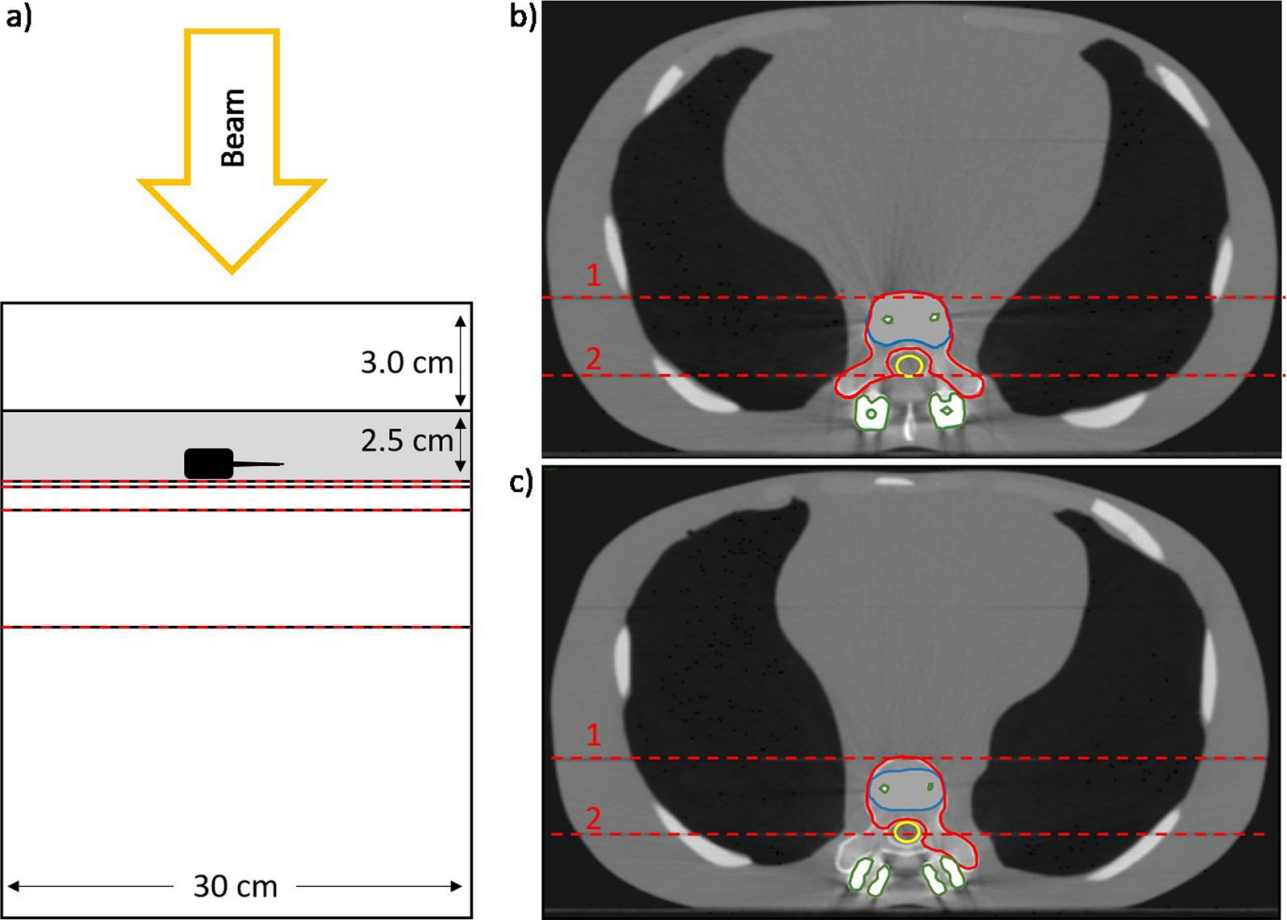
**a** A schematic view of the slab phantom with the epoxy slab in grey and the CFP-T implant in black. **b**, **c** The contoured PTVs of the anthropomorphic phantom (red), the spinal cord (yellow), the high density components (green) and bony areas (blue) for PTV1 (**b**) and PTV2(**c**), respectively. The red dashed lines on all images indicate the planes where film measurements were carried out

A more realistic and complex situation is represented by an anthropomorphic torso phantom with interchangeable spine inserts (Icotec AG, Altstätten, Switzerland and CIRS, Norfolk, USA) [23] (Fig. 1right). In the present study, the spine inserts, representing normal bone structure and CFP-T implants were employed. This phantom consists of multiple coronal slabs that allow carrying out measurements at different planes. Measurements were performed at two different planes in proximity to the CFP-T implant. These planes are indicated and labelled in Fig. 2.

Furthermore, planning-CT data sets were available at our institution for two patients with CFP-T implants, who were irradiated postoperatively with cRT and had both pre- and post-operative MRI examinations.

### CT and contouring

CT data sets were acquired for all phantoms as well as for real patients using a Brilliance Big Bore device (Philips, Netherland). Slice thickness was set to 1 and 3 mm for phantoms and patients, respectively.

All artifacts as well as the high-density titanium parts were contoured and the following densities were assigned to the corresponding regions: 1 g/cm^3^ for normal soft tissue, 1.29 g/cm^3^ for bone and 4.45 g/cm^3^ for titanium.

For the anthropomorphic phantom, two clinically realistic planning target volumes (PTVs) were delineated with consideration of preoperative tumor infiltration, namely PTV1 and PTV2. While the PTV1 encompasses the vertebral body, pedicles and both transverse processes, PTV2 is only confined to vertebral body, unilateral pedicle and left transverse process (Fig. 2). In addition, the spinal cord was contoured as OAR.

The planning-CTs, pre- and post-operative spinal MRIs from two patient cases were imported into the treatment planning software Precision (Accuray, Sunnyvale, US) and fused together. The delineation of target volumes on the planning-CTs of patients was done according to consensus contouring guidelines for post-operative SBRT [24]. Briefly, the clinical target volume (CTV) includes the gross residual tumor on postoperative imaging modalities, adjacent anatomical components of the vertebra that are at risk of microscopical spread, areas with preoperative tumor involvement and finally stabilizing implants if the risk of involvement is high [24]. The adjacent relevant OARs are contoured and spinal cord planning risk volume (PRV) was generated with 2 mm expansion. PTV was created adding 2 mm margin to CTV in all directions and cropped from spinal cord PRV in order to respect the dose constraints.

### Treatment planning and dosimetry

To assess the dosimetric accuracy of the treatment planning system, it is essential to verify that the calculated dose corresponds to the actually delivered dose. For dose delivery, the CK system employs three different beam collimator types, namely fixed collimators, Iris collimator and a multileaf collimator (MLC) [25]. In this work the Monte Carlo (MC) dose calculation algorithm, which is available for all collimator types, is used. Statistical uncertainties were set at 1% for all calculations. For dosimetric comparisons, treatment plans with different collimator types, namely Iris and MLC, are considered. In order to provide dosimetric measurements with a high spatial resolution, film dosimetry using Gafchromic EBT3 films (Ashland, US) was carried out.

Treatment plans with a single perpendicular beam impinging on the slab phantom were created for the Iris collimator and the MLC separately. For both collimators the maximal field size was used. While the 115 × 100 mm^2^ field size of the MLC allows covering the whole CFP-T implant, the 60 mm diameter Iris collimated field was centered on the densest part of the screw.

For the anthropomorphic phantom with the insert representing the CFP-T implant, treatment plans were generated using the Iris collimator and the MLC collimator for both PTVs separately. For this indication, the treatment scheme consists of delivering 24 Gy in three fractions, prescribed to the 80% isodose line. The four plans were applied and for each delivery, film measurements were carried out at the indicated positions (Fig. 2 in Section A). The same set of four plans was also applied on the anthropomorphic phantom with the spine insert and the same measurements were carried out as described above.

All irradiated films were digitized using an 10000XL (Epson, JP) scanner, corrected for lateral response artifacts of the scanner [26] and compared with the calculated dose distributions within the software FilmQA Pro (Ashland, US). In order to convert the grey values on the film into dose values, calibration stripes with a known applied dose were used to carry out a triple channel calibration [26] within FilmQA Pro. The subsequent comparisons were carried out using the green color channel.

A gamma evaluation with a dose difference criterion of 5% of the global maximal dose, a distance to agreement criterion of 1 mm and a 20% dose threshold was carried out in order to compare the calculated with the measured dose distribution.

In order to better visualize the differences between calculated and measured dose, the measured two-dimensional dose distributions were exported from FilmQA Pro and compared to the calculated dose values using python 3.6 [27].

### IGRT and delivery

The CK employs a matching algorithm that matches an orthogonal kV image pair (actual position of the patient/phantom) to the reference planning CT (2D-3D match) resulting in a correction for the setup error in 6D (translational and rotational setup errors). Inter- and intra-fraction matching is done automatically; this procedure puts a high demand on high quality CT and planar images.

In order to evaluate the accuracy of the IGRT also in the presence of the CFP-T implant, a standard machine QA test [28] was modified. For this standard machine QA end-to-end test spherically shaped dose distributions are delivered to a cube containing two orthogonal gafchromic films. This is part of the standard machine QA of the CK. On both films, the deviation of the circular isodose lines from the intended positions are registered as targeting error using the E2E-software (Accuray, Sunnyvale, US).

This cube is now affixed to the anthropomorphic phantom in the modified machine QA test and for positioning the spine match was used in the area of the phantom where the CFP-T implants are positioned. The resulting targeting error was then compared to the results of the standard machine QA test.

## Results

### CT and contouring

**Fig. 3:**
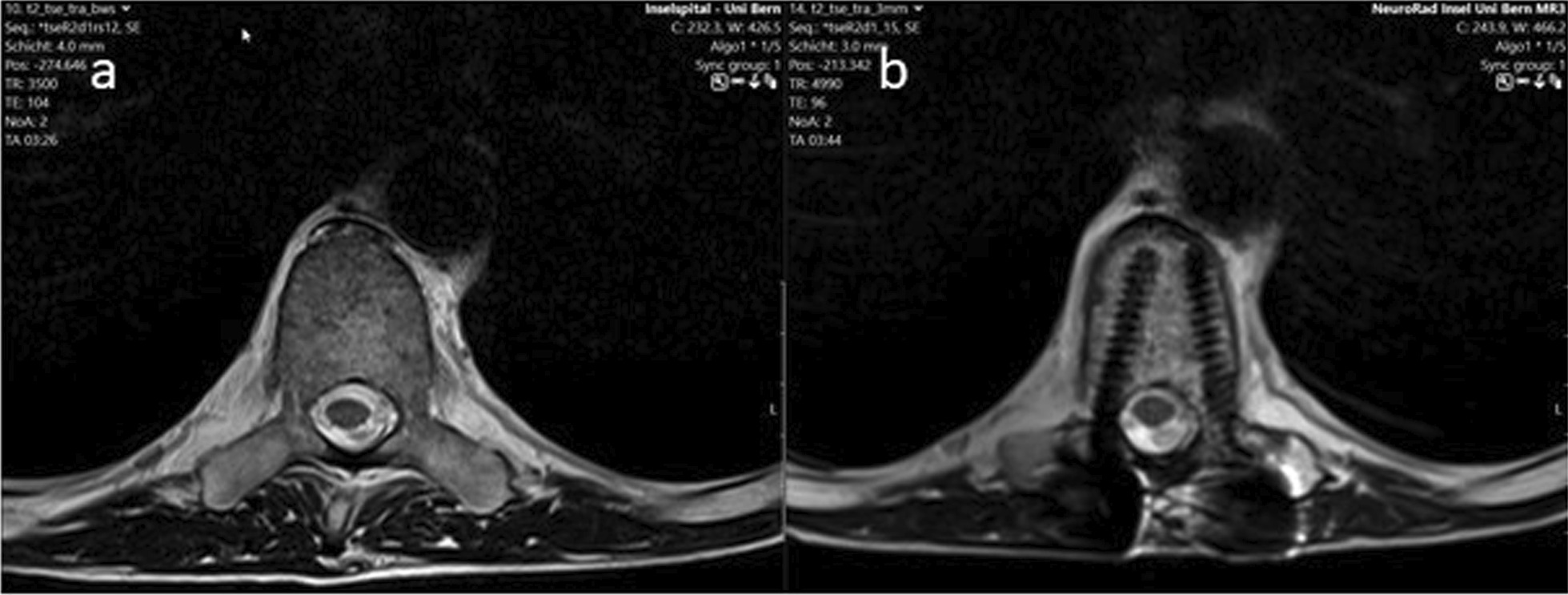
Pre- (**a**) and postoperative (**b**) T2 MRI-sequences after stabilization with the CFP-T implants. As shown on **b**, the spinal cord could be clearly visualized in the proximity of the implants

Despite artifacts from CFP-T implants, the spinal cord could be precisely visualized on T2-weighted MRI sequences and contoured (Fig. 3). In comparison to SBRT for de novo spinal metastases, there was no need for additional MRI sequences or extra PTV margins. Thus, the influence of artefacts due to the CFP-T implant was not critical.

### Treatment planning and dosimetry

#### Slab phantom

The comparison of the calculated dose distributions and the measured two-dimensional dose distributions at different distances distal to the implant using film dosimetry showed that between 95% (plane nearest to the implant) and 99% (plane 50 mm distal distance to the implant) of the voxels showed dose differences less than 5% of the dose maximum.

The maximum dose difference between the calculated and measured dose distributions was lower than 11% for all evaluated voxels.

**Fig. 4:**
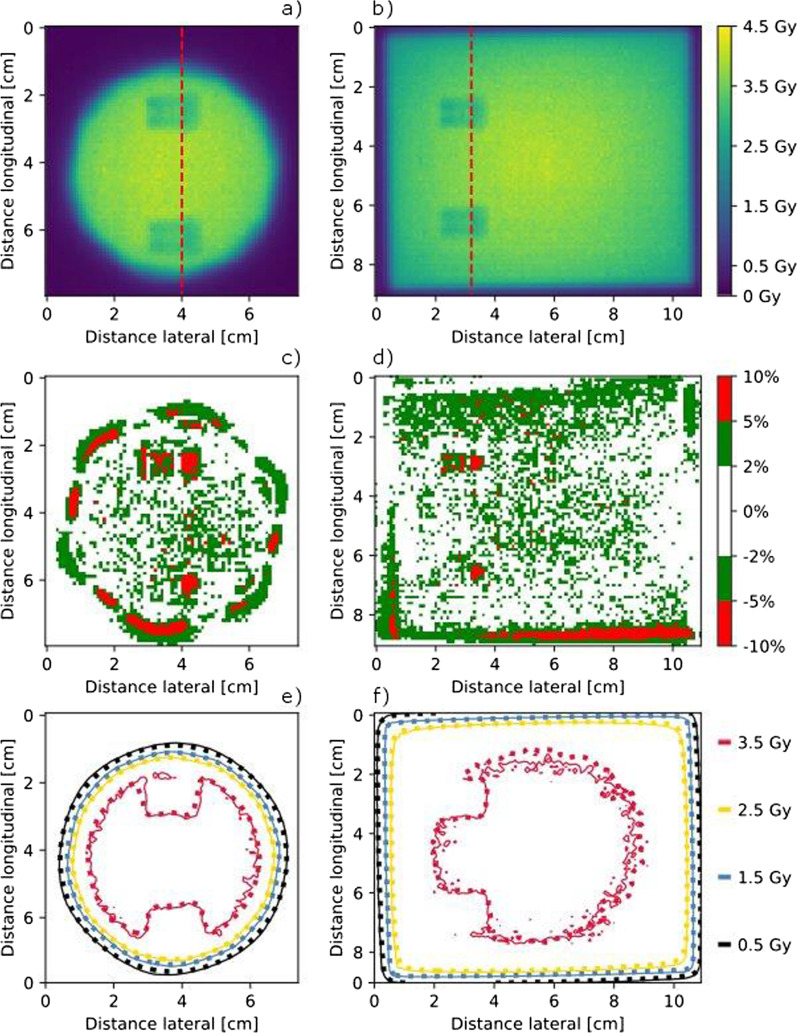
Film measured dose distribution in the measurement plane directly adjacent to the CFP-T implant, as shown in Fig. 2a, for the Iris and MLC collimated beam in **a**, **b**, respectively. Dose difference between the measured and calculated dose distribution in **c**, **d** for the Iris and the MLC collimated beam, respectively. Isodose lines of the measured (solid lines) and calculated (dotted) dose distribution in **e**, **f** for the Iris and the MLC collimated beam, respectively

**Fig. 5:**
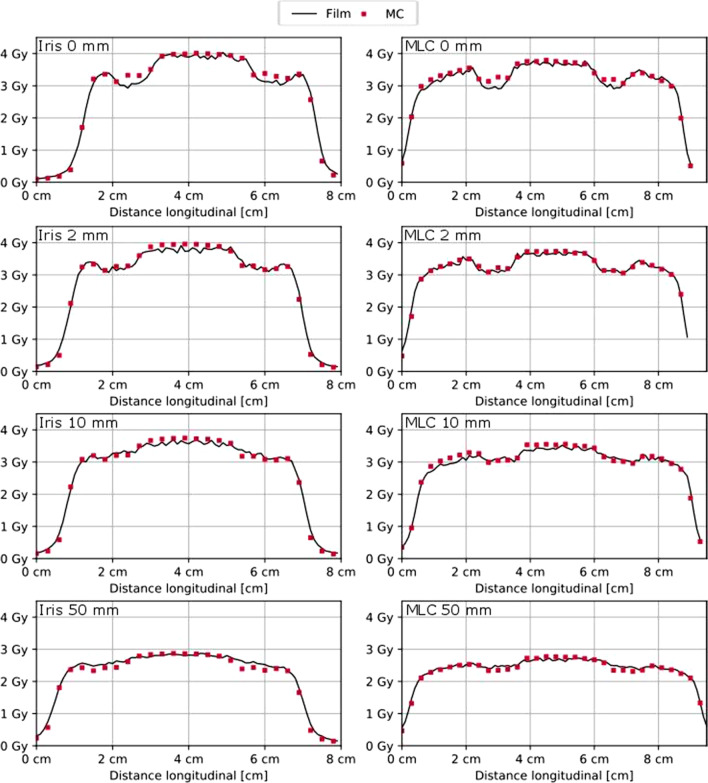
Dose Profiles (Iris left and MLC right) for all measurement planes (0, 2, 10 and 50 mm distal to the CFP-T implant) within the slab phantom as indicated in Fig. 2a by the red dashed lines. Distal distance from the CFP-T implant as well as used collimation device are indicated in each subfigure

The dose difference plots in Fig. 4 show, that the largest dose differences occur distal to the high-density parts of the screws and in the penumbra region. How well the penumbra regions match is shown by means of the isodose lines in Fig. 4e, f as well as Fig. 5. The measured and calculated dose profiles in Fig. 5 show, that the largest dose differences are present in the plane directly behind the screw, where the calculated dose values are higher than those achieved with measurements. However, for the more distal planes, the calculation seems to underestimate the dose behind the screw.

#### Anthropomorphic phantom

The gamma passing rates (5% dose maximum, 1 mm distance to agreement, 20% dose threshold) of the comparisons between the measured and calculated dose distributions are summarized in Table 1. A passing rate above 90% is considered as acceptable in clinical routine at our institution, where the measurement and calculations are carried out in a homogenous phantom. This passing rate is well achieved for all comparisons, even within the challenging heterogeneous situation.

**Table1:** Gamma passing rates of the comparison between measured and calculated dose distributions for the anthropomorphic phantom

	Collimator	Phantom	PTV 1/PTV 2
Plane 1	Iris	Bone	100%/97%
		CFP-T implant	100%/97%
	MLC	Bone	99%/97%
		CFP-T implant	100%/99%
Plane 2	Iris	Bone	99%/97%
		CFP-T implant	100%/99%
	MLC	Bone	99%/97%
		CFP-T implant	98%/97%

The position of the measurement planes is indicated in Fig. 2

**Fig. 6:**
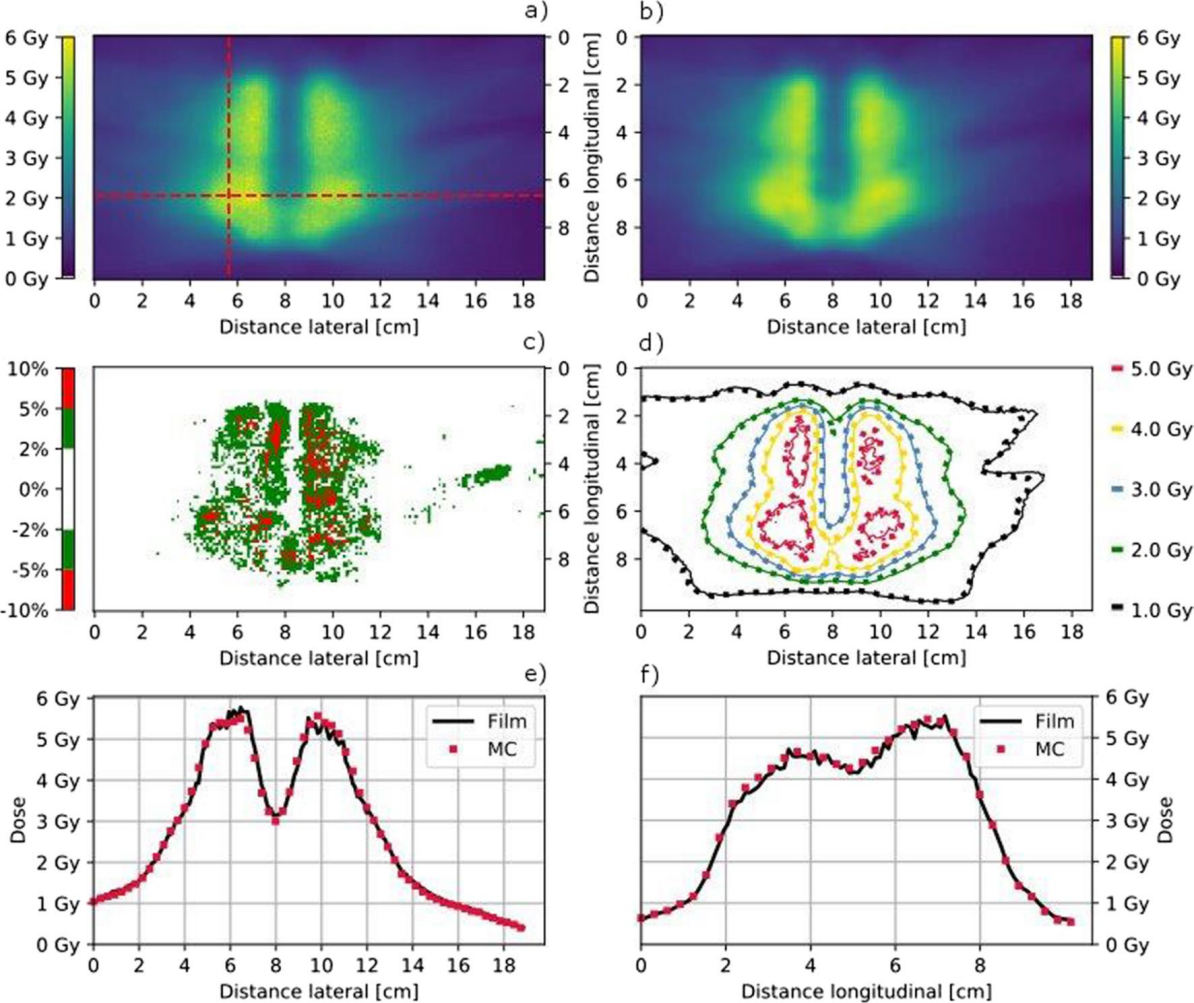
Film measured (**a**) and MC calculated (**b**) coronal dose distribution in Plane 2 for the PTV1 as indicated in Fig. 2b using the MLC collimator and the anthropomorphic phantom with the CFP-T implant. The dose difference (Film-MC) is shown in **c** and an overlay of the isodose lines (MC = dotted) is shown in **c**, **d**, respectively. Horizontal (**e**) and transversal (**f**) dose profiles are indicated in **a**

**Fig. 7:**
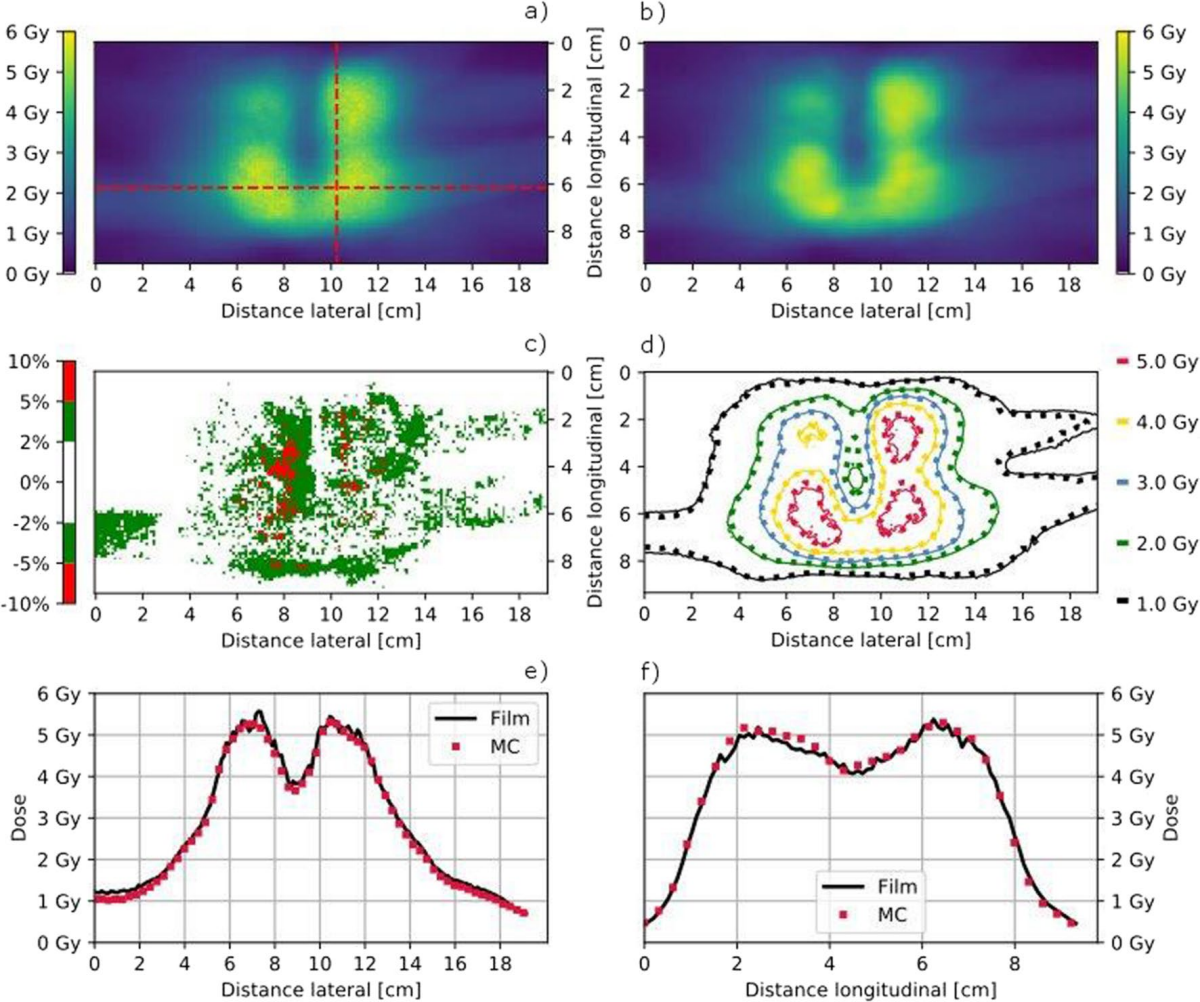
Film measured (**a**) and MC calculated (**b**) coronal dose distribution in Plane 2 for the PTV2 in the posterior plane indicated in Fig. 2c using the Iris collimator and the anthropomorphic phantom with the CFP-T implant. The dose difference (Film-MC) is shown in **c** and an overlay of the isodose lines (MC = dotted) is shown in **d**. Horizontal (**e**) and transversal (**f**) dose profiles are indicated in **a**

For the anthropomorphic phantom equipped with the CFP-T implant, the calculated and measured dose distributions in the plane 2 are visualized in Figs. 6 and 7. Only few regions surpass a dose difference of 5%. The isodose line comparison as well as the plotted profiles through the high dose region show an excellent agreement between measurements and calculations.

### IGRT and delivery

**Fig. 8:**
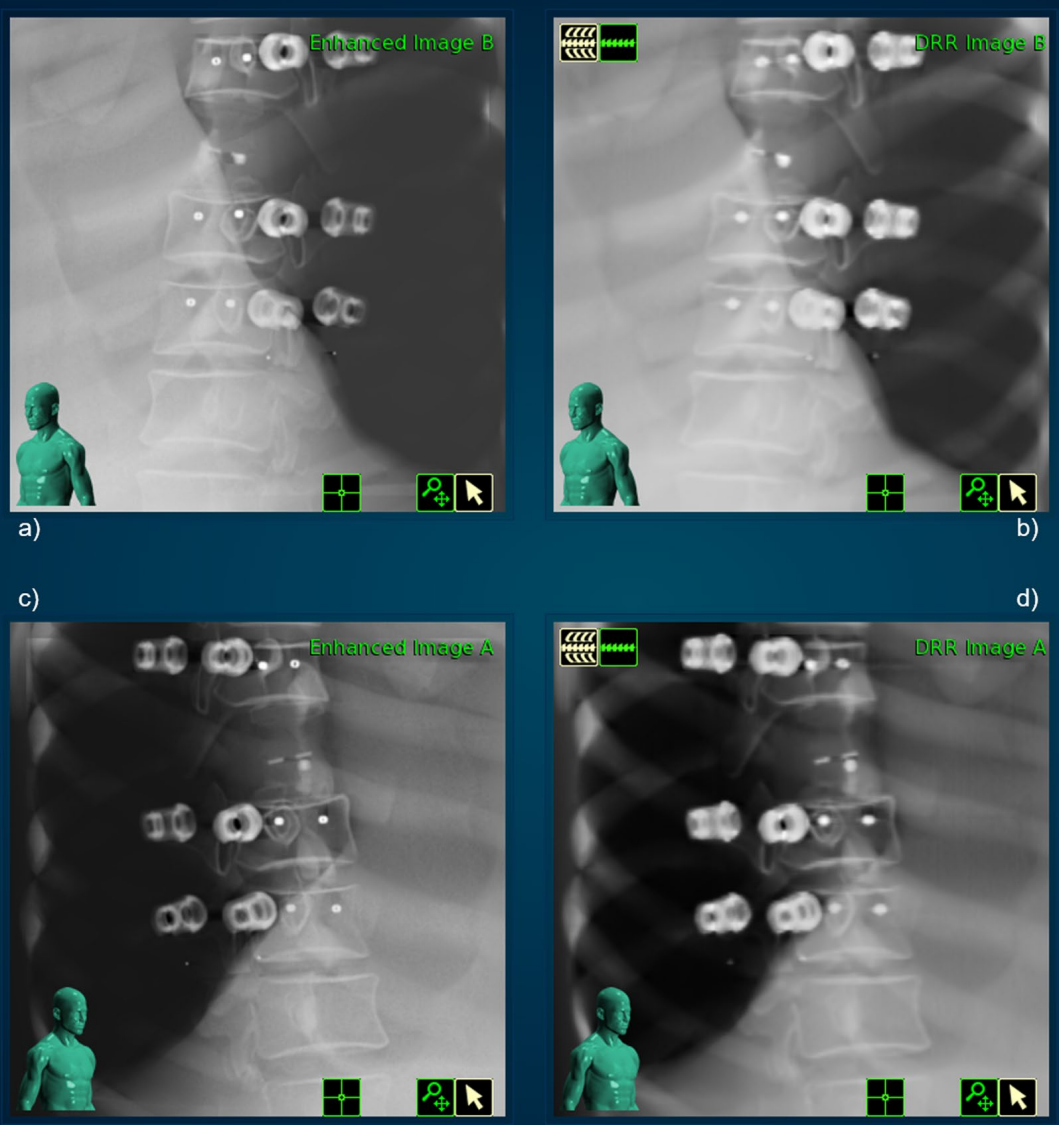
Live image for both x-ray cameras on **a**, **c** as well as the corresponding calculated DDR images on **b**, **d**

The acquired images within the treatment delivery procedure are shown in Fig. 8. The digital reconstructed radiograph (DRR) as well as the orthogonal kV images do not show much deterioration. The quantitative evaluation of the IGRT process, as described in the Materials and Methods section leads to the following results: The targeting error was 0.71 mm, which is in accordance to the achieved accuracy in regular machine QA and well below the actual threshold for those tests (0.95 mm) as recommended by S. Dieterich et al. [29].

## Discussion

In the present study, we demonstrate the feasibility of spine SBRT in proximity of CFP-T hybrid implants using Cyberknife.

Besides MESCC, spinal instability is a routine indication for surgical management of metastatic spine. The spine instability neoplastic score (SINS-Score) is a comprehensive classification system and helps the clinicians to select patients who might benefit from a surgical stabilization [30].

Until now, only one prospective phase II trial reported the results for postoperative spinal SBRT [16]. With a median follow-up of 10.5 months, 33 treated patients achieved radiographic and symptomatic excellent local control of 90% at 1 year with low toxicity profile. The authors mentioned the occasional difficulties regarding imaging artifacts caused by spinal metal implants, which made the use of CT-myelogram inevitable to visualize spinal cord.

Postoperative, SBRT has been delivered to tumor sites with metal hardware, traditionally made of titanium [18]. The metal implants are high-Z materials and although offer good stability and stiffness, they can affect the dose distribution to target and normal tissues due to the scattering effects of ionizing radiation [31]. Furthermore, hardware failure is a relevant issue after spine surgery, which could be partly due to irradiation [32]. According to consensus guidelines for postoperative SBRT for spinal metastases, there is no need to include surgical instruments and incision in the CTV [33]. Therefore, someone can hypothesize that SBRT might reduce the rate of hardware failure compared to conventional RT. Unfortunately, there are sparse data regarding type of implants and hardware failure in studies with postoperative spine SBRT.

To overcome those problems with metal hardware, CFP implants have been developed and widely used in spine surgery [34]. CFP cages are biocompatible, promote bone fusion and have been used in surgical interventions since many years [35, 36]. With further developments, spinal fixation system (rods and screws) was introduced, totally or partially made of CFP. The biomechanical studies showed even benefits of CFP implants in terms of reducing risk of adjacent segment disease (ASD) and hardware failure when compared to titanium fixation system [37]. Considering the stiffness and resistance to motion, CFP implants are equal to titanium and better than pure polyetheretherketone (PEEK) [38].

Tedesco et al. reported the results of 22 patients with primary spine tumors, who underwent surgical interventions using composite CFP fixation system [39]. Unfortunately, there are no details about the type of RT and authors concluded that CFP implants are comparable to titanium regarding complications, stability at weight bearing and functional recovery [39]. In another study from the same group, a mixed cohort including 20 primary and 14 metastatic spine tumor patients were treated with CFP composite implants [40]. All cases had postoperative RT. Again, there is no reported data about type and intention of RT. With a mean follow up of 13 months (6–36 months), only two cases (6%) had hardware failure due to local recurrence, the overall rate of local failure was 17.6% (6/34) [40].

In 2017, Ringel et al. published a study consisting of 35 patients, mostly with spinal metastasis, after posterior stabilization using CFP-T pedicle screws (Icotec, Altstätten, Switzerland) [41]. The study evaluated the feasibility of CFP-T fixation system and their impact on postoperative imaging as well as radiotherapy planning. Of interest, RT plans were made for both photons and protons. Matched controls with titanium were used to assess the absorption of ionizing radiation and imaging artifacts in patients with CFP-T implants. Almost all screws (1 revision out of 251) were placed correctly through the pedicles as seen on postoperative imaging. For RT treatment plans, notably smaller CT-Hounsfield values for CFP-T implants compared to titanium screws improved the precision of dose calculation, especially for proton beams. The CFP-T screws used in above study are titanium-coated in the pedicle part to improve osseointegration and better fusion to the bone, as it was shown in a prospective comparative study between titanium-coated and uncoated PEEK cages in lumbar surgery [42].

In our study, we assessed the essential steps in RT workflow for postoperative spine SBRT in proximity of CFP-T hybrid implants using a Cyberknife. The fusion of patients` pre- and post-operative MRI-sequences to the corresponding planning CTs, as well as target delineations could be carried out with the same accuracy as for cases with de novo spinal metastases. The contouring was done, according to recommendations from international consensus guidelines for postoperative spine SBRT [24]. As epidural space is the most common site of treatment failure after postoperative SBRT [43], we contoured the true spinal cord instead of thecal sac to avoid under-dosage and improve LC. There was no need for enlarged CTV-to-PTV margins due to the presence of CFP-T implants.

For treatment planning, including dose calculation, a density correction was applied to the high-density parts as well as the artifacts. Technical solutions like dual energy CT, which might reduce the artifacts are not clinical standard at our institution. Thus, the application of such technologies is beyond the scope of this work but will be evaluated in future studies.

For the single beam impinging on the slab phantom with the CFP-T implant present 95% of the voxels show a difference < 5% between the measured and calculated dose distributions and a maximal dose difference of 11%. Regarding the multitude of non-coplanar beams present in a Cyberknife treatment plan, hence a mitigation of the magnitude of the dose differences is expected in patient treatment plans.

The very high gamma passing rates for the anthropomorphic phantom show that almost all voxels with a dose differences larger than 5% are within 1 mm distance to agreement that is regions where high dose gradients are present. The situation with the spine insert was used as comparison. The results of the gamma analysis and the spatial distribution of the dose differences for both situations were similar.

The IGRT including the automatic matching algorithm did not show any issues throughout all executed tests. Convincing were also the results of the modified machine QA test, which were in the range of the regular standard tests and well within the recommended thresholds [29].

## Conclusion

Based on the results of this study, we have shown the dosimetric and delivery feasibility of postoperative spine SBRT in proximity of CFP-T implants using a CyberKnife machine.

## Data Availability

Not applicable.

## Abbreviations

SBRT: Stereotactic body radiation therapy
CFP-T: Carbon fiber reinforced polyetheretherketone and titanium
RT: Radiation therapy
OAR: Organ at risk
IGRT: Image guided radiation therapy
QA: Quality assurance
MESCC: Malignant epidural spinal cord compression
VCF: Vertebral compression fracture
cRT: Conventional RT
LC: Local control
HRQOL: Health related quality of life
CT: Computer tomography
CK: CyberKnife
PTV: Planning target volume
CTV: Clinical target volume
PRV: Planning risk volume
MLC: Multileaf collimator
MC: Monte Carlo
2D: Two dimensional
DRR: Digital reconstructed radiograph
SINS: Spine instability neoplastic score
ASD: Adjacent segment disease
PEEK: Polyetheretherketone
